# Deletions in the Y-derived amelogenin gene fragment in the Indian population

**DOI:** 10.1186/1471-2350-7-37

**Published:** 2006-04-10

**Authors:** VK Kashyap, Sanghamitra Sahoo, T Sitalaximi, R Trivedi

**Affiliations:** 1 National DNA Analysis Centre.Central Forensic Science Laboratory, Kolkata, INDIA; 2 National Institute of Biologicals, A-32, Sector 62, Institutional Area, Noida 201307, Uttar Pradesh, India

## Abstract

**Background:**

Rare failures in amelogenin-based gender typing of individuals have been observed globally. In this study, we report the deletion of a large fragment of the amelogenin gene in 10 individuals out of 4,257 male samples analyzed from 104 different endogamous populations of India.

**Methods:**

Samples were analyzed using commercial genetic profiling kits. Those that exhibited failures in amelogenin-based gender identification were further analyzed with published as well as newly designed primers to ascertain the nature and extent of mutation.

**Results:**

The failure rate among Indian males was 0.23 %. Though the exact size and nature of the deletion (single point mutations at a number of positions or a single large deletion) could not be determined in the present study, it is inferred that the deletion spans a region downstream of the reverse primer-binding site of commercially available amelogenin primer sets. Deletions were conspicuously absent among the Mongoloid tribes of Northeast India, while both caste and tribal groups harbored these mutations, which was predominantly among the Y-chromosomes belonging to J2 lineage.

**Conclusion:**

Our study indicates that the different amelogenin primer sets currently included in genetic profiling multiplex kits may result in erroneous interpretations due to mutations undetectable during routine testing. Further there are indications that these mutations could possibly be lineage-specific, inherited deletions.

## Background

Genotyping the X-Y homologous amelogenin gene segment for gender identification is widely used for DNA profiling in DNA databasing, forensic casework, archeological specimens, preimplantation and prenatal diagnoses [1-4]. The amelogenin gene is a single copy gene, homologues of which are located on Xp22.1-Xp22.3 and Yp 11.2 [5]. Regions on this gene that are sufficiently conserved are amplified for simultaneous detection of the X and Y alleles in gender identification procedures. Primers bind to the first intron region of the amelogenin gene on the X and Y-chromosomes [6] and amplify regions that differ in base sequence, hence resulting in products that are easily distinguishable by differences in size and sequence. The most widely used primer set [6] delimits a 6 bp deletion on the X-chromosome and produces fragments of 106 bp and 112 bp for the X and Y chromosomes respectively. Presence of two amplified products indicates a male genotype, while a single amplicon implies female genotype. However, mutations in the Y-derived fragment of the gene may result in amplification failure of the Y-allele, causing misidentification of the biological sample as of a female. Similarly, mutations on the X homologue would also result in non-amplification of the X-derived fragment although the genotype would still be identified as male due to amplification of the Y-amelogenin allele.

Recently, a few studies have revealed misidentification of the male genotype while employing the amelogenin gender test [[7-9]]. Failures in accurate determination of gender have been reported to be particularly high among individuals of Indian origin. The frequency of failure was observed to be 8 % by Santos et al. [7], while Thangaraj et al. [9] reported 5 cases of amelogenin failure (1.85 %) among the 270 Indian males studied. The failure rate of the amelogenin sex test was particularly high (3.6 %) in an Indian population group from Malaysia [10]. However, a parallel study testing a larger number of individuals from the Austrian National DNA database reported a failure rate of 0.018 % [11]. The high frequency in incidence of failures in the Indian sub-continent prompted us to scrutinize the amelogenin typing results of 7,214 individuals (including 4,257 males) belonging to 104 different endogamous populations that were genotyped as part of our DNA databasing project. Individuals were sampled from diverse geographic regions across India such that all existing socio-ethnic groups and linguistic families were represented. In this paper, we report failures in genotyping of male individuals due to mutations originating in the Y-homologue of the amelogenin gene. We have further characterized the nature and extent of mutations and provide evidence for a plausible inherited mode of transmission of the mutation.

## Methods

### Population samples analyzed

**Figure 1:**
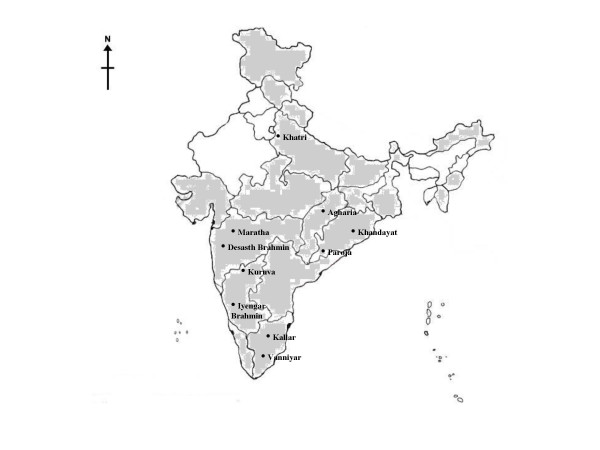
Map depicting the regions covered in the study and location of samples where amelogenin deletion were observed.

DNA was isolated by standard organic extraction method [12] either from blood or buccal swabs of consenting 4,257 male and 2,957 female individuals belonging to 104 different endogamous groups. Individuals represented major caste and tribal groups of India, which were sampled from across 20 geographical regions of India (Table 1, Figure 1).

**Table 1:** Incidences in failures of gender testing among the 104 endogamous populations of India tested for amelogerin gene efficacy

**S.No**	**State**	**Population**	**Social Group**	**Individuals Tested**	**Failure in Gender Analysis (No of samples)**
1	Jammu & kashmir	**Balti**	Tribe	67	Nil
		**Drokpa**	Tribe	38	Nil
		**Argon**	Tribe	51	Nil
		**Buddhist**	Religious Group	156	Nil
2	Himachal Pradesh	**HPRajput**	Caste	50	Nil
3	Uttaranchal	**Tharu**	Tribe	45	Nil
		**Jaunsari**	Tribe	45	Nil
		**Bhoksha**	Tribe	42	Nil
4	Uttar Pradesh	**Kanyakubj Brahmin**	Caste	98	Nil
		**UP Jat**	Caste	48	Nil
		**UP Thakur**	Caste	48	Nil
		**Khatri**	Caste	47	**1**
		**UP Kurmi**	Caste	45	Nil
5	Bihar	**Bihar Brahmin**	Caste	59	Nil
		**Bhumihar**	Caste	65	Nil
		**Rajput**	Caste	58	Nil
		**Kayasth**	Caste	53	Nil
		**Yadav**	Caste	44	Nil
		**Kurmi**	Caste	50	Nil
		**Baniya**	Caste	45	Nil
6	Gujarat	**Gujarat Patel**	Caste	45	Nil
7	Maharastra	**Desasth Brahmin**	Caste	70	**1**
		**Chitpavan Brahmin**	Caste	78	Nil
		**Maratha**	Caste	65	**1**
		**Dhangar**	Caste	150	Nil
		**Pawara**	Tribe	82	Nil
		**Katkari**	Tribe	95	Nil
		**Madia Gond**	Tribe	45	Nil
		**Mahadeo Koli**	Tribe	45	Nil
8	Chattisgarh	**Brahmin**	Caste	51	Nil
		**Satnami**	Caste	50	Nil
		**Teli**	Caste	50	Nil
		**Dheria Gond**	Tribe	35	Nil
		**Agharia**	Tribe	70	**1**
		**Oroan**	Tribe	42	Nil
9	Jharkhand	**Ho**	Tribe	50	Nil
		**Bhumij**	Tribe	56	Nil
		**Kharia**	Tribe	83	Nil
		**Munda**	Tribe	64	Nil
		**Birhor**	Tribe	61	Nil
		**Santhal**	Tribe	61	Nil
		**Oroan**	Tribe	60	Nil
10	Westbengal	**Brahmin**	Caste	110	Nil
		**Kayasth**	Caste	103	Nil
		**Mahishya**	Caste	60	Nil
		**Namasudra**	Caste	55	Nil
		**Bauri**	Caste	54	Nil
		**Maheli**	Tribe	49	Nil
		**Karmali**	Tribe	51	Nil
		**Kora**	Tribe	59	Nil
		**Lodha**	Tribe	99	Nil
11	Orrisa	**Oriya Brahmin**	Caste	57	Nil
		**Karan**	Caste	62	Nil
		**Khandayat**	Caste	62	**1**
		**Gope**	Caste	60	Nil
		**Paroja**	Tribe	78	**1**
		**Juang **	Tribe	50	Nil
		**Saora**	Tribe	35	Nil
12	Andhra Pradesh	**Andhra Brahmin**	Caste	106	Nil
		**Raju**	Caste	66	Nil
		**Kappu Naidu**	Caste	104	Nil
		**Kamma Chaudhary**	Caste	106	Nil
		**Reddy**	Caste	107	Nil
		**Komati**	Caste	104	Nil
		**Yerukula**	Tribe	101	Nil
		**Chenchu**	Tribe	100	Nil
		**Naikpod Gond**	Tribe	104	Nil
		**Lambadi**	Tribe	107	Nil
		**Golla**	Caste	65	Nil
		**Sakunupakshollu**	Caste	30	Nil
13	Tamil Nadu	**Chakkiliar**	Caste	49	Nil
		**Tanjore Kallar**	Caste	101	**1**
		**Vanniyar**	Caste	87	**1**
		**Pallar**	Caste	33	Nil
		**Gounder**	Caste	56	Nil
		**Irular**	Tribe	54	Nil
		**Paraiyar**	Caste	21	Nil
14	Kerala	**Nair**	Caste	87	Nil
15	Karnataka	**Iyenger Brahmin**	Caste	65	**1**
		**Lingayat**	Caste	98	Nil
		**Gowda**	Caste	56	Nil
		**Bhovi**	Caste	52	Nil
		**Christian**	Religious Group	55	Nil
		**Muslim**	Religious Group	65	Nil
		**Kuruva**	Tribe	60	**1**
16	Sikkim	**Bhutia**	Tribe	75	Nil
		**Nepali**	Caste	110	Nil
		**Lepcha**	Tribe	48	Nil
17	Mizoram	**Mara**	Tribe	90	Nil
		**Hmar**	Tribe	80	Nil
		**Lai**	Tribe	92	Nil
		**Lusei**	Tribe	92	Nil
		**Kuki**	Tribe	105	Nil
18	Arunachal Pradesh	**Adi Pasi**	Tribe	203	Nil
19	Manipur	**Garo**	Tribe	110	Nil
		**Meitei**	Tribe	105	Nil
		**Naga**	Tribe	106	Nil
		**Hmar**	Tribe	101	Nil
		**Manipuri Muslim**	Religious Group	101	Nil
20	Andaman & Nicobar Islands	**Great Andamanese**	Tribe	24	Nil
		**Jarawa**	Tribe	50	Nil
		**Onge**	Tribe	16	Nil
		**Nicobarese**	Tribe	28	Nil
		**Shompen**	Tribe	33	Nil

* NUMBER OF POPULATIONS ANALYZED: 104; 4257 MALES OUT OF 7214 INDIVIDUALS ANALYZED IN GENETIC PROFILING

### Amelogenin typing using commercial genotyping kits

The DNA samples were amplified using commercial multiplex short tandem repeat (STR) kits; PowerPlex^® ^16 system (Promega Corporation, Madison, USA) and Identifiler™ (Applied Biosystems, Foster City, CA), which include the amelogenin marker for gender determination. Genotyping of the amplified products was performed on an ABI Prism™ 377 DNA Sequencer (PE Applied Biosystems, Foster City, CA). The amelogenin profile was determined from the electropherograms by comparing the presence or absence of 106 and 112 bp peaks with known male and female controls. Females exhibit a single peak of 106 bp while males exhibit two peaks of 106 and 112 bps.

### Amelogenin typing using newly designed primers and other published primers

Samples that showed abnormal amelogenin peak profiles with the commercial kits were reamplified with primers described by Steinlechner et al. [11] followed by genotyping as described above. Male samples exhibit two peaks of 219 and 225 bp, while female samples exhibit a single peak at 219 bp.

Additional primers were designed for amplifying the region identified by Roffey et al. [8] to decipher the origin of mutations. The sequences of new primers designed to facilitate detection of mutation are as follows:

1. #P1: 5'- TTACGGCCATATTTAGGA-3' (for amplification of X and Y homologues)

2. #P2: 5'- GAAAGAGTCAATCCGAATGGT-3' (for amplification of Y homologue)

### Analysis of SRY, Y-STRs and Y-SNPs

To confirm the gender of the studied samples, a sex-determining locus (SRY) [13] specific to males, was amplified. Occurrence of a single 93 bp amplicon would distinguish an authentic male DNA sample from a female DNA sample. Further, four Y- short tandem repeats (Y-STRs) [14,15] (DYS19, DYS389I, DYS389II and DYS390) were amplified to determine the extent of deletion of the Y-chromosome and to determine if Y-STR haplotype profiles were shared between individuals. Y-STR amplification was carried out in a single tube multiplex reaction [14] and genotyped on an ABI Prism™ 377 DNA Sequencer (PE Applied Biosystems, Foster City, CA). Y-single nucleotide polymorphisms (Y-SNPs) (M89, M9, M172, 92R7, M45, M20, M70, M214, M69, M124, M173, M17) [16] were profiled hierarchically to identify the lineage of the test samples.

## Results and Discussion

Out of the 4,257 males analyzed with either PowerPlex^® ^16 or Identifiler™ multiplex system, 10 confirmed male samples exhibited a dropout of the 112 bp amelogenin Y-allele (Table 1). To verify the cause of observed abnormalities, we tested such samples with alternate primer pairs that encompassed the region amplified with primers reported by Sullivan et al. [6] and, are also typically used in the commercial kits. Amplification of the test samples with the primer set described by Steinlechner et al. [11] resulted in complete absence of the 225 bp Y-specific product.

All 10 ambiguous samples were confirmed to be from male individuals on testing with male-specific SRY locus, which yielded the characteristic 93 bp amplicon reconfirming the gender of these subjects as males.

**Figure 2:**
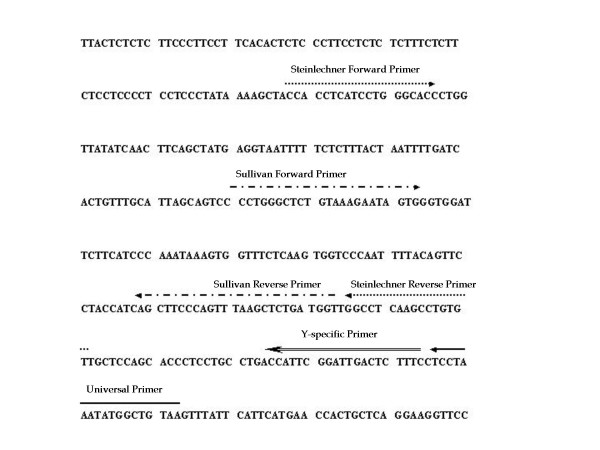
Y-chromosome nucleotide sequence of the human amelogenin gene (GenBank Accession Number M55419) showing the location of annealing regions of the primer sets used in this study.

Additional analysis with four Y-chromosomal STR markers, DYS19, DYS389I, DYS389II and DYS390, yielded complete and different Y-STR haplotype profiles. Amplification of Y-STR indicates that these samples had failed the amelogenin typing either due to mutation in the primer-binding region [17] or due to deletions in the amelogenin region (11.2p) on the Y-chromosome [11]. Among the 10 samples, eight distinct Y-STR haplotype profiles were observed; one was shared between Khatri and Kuruva and, another one by Agharia and Kallar (Table 2). Further amplification and sequencing was carried out using the forward primer of Steinlechner et al. [11] and a set of newly designed reverse primers spanning the hypothetical region of mutation – #P1, which is 62 bp downstream, and #P2, a Y-specific primer that is 43 bp downstream to the Steinlechner's reverse primer binding region, in order to determine the nature and extent of mutation. The newly designed primers depicted in Fig.2, result in 287 bp and 268 bp amplicon for #P1 and #P2 respectively, for the Y-chromosome, when used along with the forward primer of Steinlechner et al., #P1 results in a 281 bp product for the X-chromosome. However, the newly designed and validated primer sets also failed to amplify the Y-homologue in test samples suggesting deletion of a significant portion of the amelogenin region in male samples. The deletion in amelogenin gene has recently been mapped to span around 2.5 Mb [18].

**Table 2:** Y-chromosome profiles of the amelogenin-deletion individuals*

*S. No*	*Sample*	*Y-SNP*	*DYS 19*	*DYS 389I*	*DYS 389II*	*DYS 390*
1	Kallar	NA	**15**	**12**	**29**	**25**
2	Vanniyar	J2	15	13	29	24
3	Agharia	NA	**15**	**12**	**29**	**25**
4	Khatri	NA	***15***	***13***	***30***	***25***
5	Iyenger Brahmin	J2	14	13	29	24
6	Kuruva	NA	***15***	***13***	***30***	***25***
7	Khandayat	J2	15	11	30	23
8	Paroja	J2	15	13	26	25
9	Desasth Brahmin	J2	14	13	30	23
10	Maratha	J2	15	11	28	25

NA: Samples not be examined for Y-SNPs

* Integers in bold represent shared haplotype between Kallar and Agahria; while those in bold italics represent haploype sharing between Khatri and Kuruva

The overall rate of failure among the Indian population was found to be 0.23%. Table 1 shows the frequency of failure among different endogamous groups. Failures were exhibited by both caste groups (Khatri, Desasth Brahmin, Maratha, Khandayat, Tanjore Kallar, Vanniyar and Iyenger Brahmin) and tribal populations (Agharia, Paroja, and Kuruva) while Mongoloid and Negrito populations were not found to harbor the deletions (Fig. 1). Interestingly, we observed that these deletions were present predominantly in individuals belonging to the J2 Y-chromosomal lineage. J2 is found in approximately 5.1% of the Indian population, while majority of the Indian males harbor H (25%), R1a1 (19%) and R2 (16%) haplogroups in their Y-chromosomes [19]. Probably originating in the Middle East [20], the J2 lineage has been found distributed across southeastern Europe and Asia with frequencies of 6.5% in Central Asians [16], 23.8% in Sephardic Jews, 20% in Lebanese, 17.8% in Konyan Turks, 16.3% among Italians of Apulia, 13.6% in French Basque [20], 10.2% in Moroccan Arabs [21]. The above observations and positive amplification of the SRY gene and the appearance of discrete Y-STR haplotypes, suggests that the mutations probably arose independently on a J2 Y chromosome lineage background.

Of the endogamous populations screened in this study, ~10 % exhibited failures in the amelogenin gender test. Since the extent of deletion is large to avoid amplification dropout of the Y-homologue with currently available commercial primer sets, we suggest it would be prudent to include an additional gender test such as SRY and/or Y-STR testing for accurate gender identification of biological specimens.

## Conclusion

Earlier studies have reported high failure rates in amelogenin-based gender testing of individuals from the Indian sub-continent. In this study, we have analyzed 4,257 male samples and report a failure rate of 0.23%. Due to ease of typing, this test has gained wide acceptance and has been integrated into routine automated genetic profiling procedures. However, the fallibility of the amelogenin test raises concern over its continued use especially in medical and forensic sciences.

Although our study indicates that individuals belonging to the J2 lineage are more prone to deletion in Y-derived amelogenin gene, further corroborating studies are desired. The amelogenin-based gender test thus needs to be applied with caution, with supplementation with other Y-chromosome specific analyses for reliable gender identification.

## Competing interests

The author(s) declare that they have no competing interests.

## Authors' contributions

VKK conceptualized the study and contributed significantly in data interpretation and manuscript preparation. SS and TS contributed equally towards designing and carrying out of experiments, data analyses and in manuscript preparation. RT provided critical and valuable information for data processing. All authors read and approved the final manuscript.

## Pre-publication history

The pre-publication history for this paper can be accessed here:


